# The effect of rural and urban life on peritonitis rates in chronic peritoneal patients

**DOI:** 10.1080/0886022X.2022.2163504

**Published:** 2023-01-16

**Authors:** Erdem Çankaya, Murat Altunok, Aycan Mutlu Yağanoğlu

**Affiliations:** a Department of Nephrology, Medical Faculty, Atatürk University, Erzurum, Turkey; b Department of Animal Science, Faculty of Agrıculture, Atatürk University, Erzurum, Turkey

**Keywords:** Peritoneal dialysis, urban area, rural area, peritonitis rate

## Abstract

**Background:**

It has been reported that living far from the peritoneal dialysis (PD) unit is a risk factor for peritonitis. Considering that PD units are urban located; the question of whether living in a rural area compared to an urban area is a risk factor for peritonitis has arisen.

**Methods:**

From March 2010 to August 2020, 335 episodes of peritonitis in 202 PD patients followed in a single center were evaluated retrospectively. People living in areas with a population <1000 were defined as living in rural areas regardless of their distance from the PD center. Cox regression analysis was used to identify independent factors associated with peritonitis.

**Results:**

A total of 202 PD patients were followed during 791 patient-years (mean follow-up of 3.9 years per patient). Total patients had 335 episodes of peritonitis and the rate of peritonitis was 0.42 episodes per year (episodes/patient-year). Cox regression analysis revealed that living environment (urban *vs*. rural) was not a risk factor for peritonitis (*p* = 0.57).

**Conclusions:**

In Turkey, we report that living in a rural area in our region is not a risk factor for peritonitis. It is not the right approach for both the physician and the patient to be reluctant in the choice of PD due to the concern of peritonitis in rural areas.

## Introduction

Peritoneal dialysis (PD) is applied as renal replacement therapy (RRT) at a rate of approximately 11% over the world [1]. Peritonitis remains one of the most serious and life-threatening complications of PD. Besides that, ultrafiltration loss and permanent peritoneal membrane damage are closely related to the necessity of removing the catheter, which leads to technical failure and hemodialysis (HD) conversion [2].

In Turkey, 4.06% (*n*: 3387) of the patients receiving RRT perform PD. RRT starts with PD in approximately 10% (1200) of patients every year [3].

In the guideline published by the International Society of Peritoneal Dialysis (ISPD) in 2022, both the prevention and treatment of peritonitis are at the forefront. Risk factors in peritonitis associated with PD are divided into two groups non-modifiable and modifiable [4]. Living far from the PD unit in the modifiable group is one of them [5]. Considering that the PD units are located in urban areas, the question of whether living in a rural area is a risk factor for peritonitis was brought to mind. In addition, the desire to choose PD has increased due to the problems in the accessibility to HD in the rural patient group. However, the fact that living away from the PD unit increases the risk of peritonitis may cause physicians to hesitate about initiating PD in this patient group. We believe that more studies are needed; since there are few about that subject, to answer whether living away from the PD unit as well as living in rural and urban areas is a risk factor independent of distance.

## Material and methods

A total of 202 (with and without peritonitis) patients were evaluated. Between March 2010 and August 2020, 335 episodes of peritonitis in 202 patients who have undergone PD treatment and were followed up by our clinic were evaluated retrospectively. It was a single-center study. Our center was the only actively-serving center in eastern Turkey. The patients were followed up with the same nurse group (two nurses). The patients were trained regardless of the duration by the same nurses in a way that they and their assistants would perform PD, and PD was started. Approval was obtained from the local ethics committee. Since it was a retrospective observational study, patient consent was not required. Peritonitis was diagnosed according to peritoneal dialysis (ISPD) guidelines 2022 International Society, at least two of the following criteria were required: clinical features consistent with peritonitis, that was abdominal pain and/or cloudy dialysis effluent, dialysis effluent white cell count >100/mL or >0.1 × 10^9^/L (after a dwell time of at least 2 h), with >50% polymorphonuclear leukocytes (PMN), positive dialysis effluent culture [6]. Gram stain, culture, Mycobacterium Tuberculosis culture and staining, and fungal cultures were routinely requested from all patients presenting with peritonitis episodes. All patients diagnosed with peritonitis were followed up in our center until culture results were obtained. Overall peritonitis rate calculation included all peritonitis events, not just the first peritonitis event. Living in rural and urban areas was determined from addresses in the hospital registration system. Rural living areas were those living in hamlets, villages, and towns connected to districts and central districts (Figure 2). The definition of the rural area was taken as those living in areas with a population <1000. Although their distance to the PD center was not assessed, the nearest rural area to the PD center was over 30 km. The study population was ethnically homogeneous and of Caucasian descent. The peritonitis rate in urban and rural areas was calculated as the annual number of attacks (episodes per patient/years) recommended by ISPD 2016 (2). However, some of the socio-demographic (educational status, heating type of the house, marital status, number of people living at home, type of construction of the house, whether they use a separate area for PD, whether they use an assistant for PD, etc.) characteristics of the patients were also examined.

### PD modalities

At PD initiation, participants were assigned to one of the PD modalities; CAPD with a twin-bagged system or instrumented peritoneal dialysis (IPD) (6–8 h with 6–10 l). The prescription of CAPD was 4 × 1.5–2 L (body surface area, RRF determined) exchanges as long as no sign of inadequate dialysis was observed. Dialysate fluids containing (i) 1.36%, 2.27%, or 3.86% glucose; (ii) amino acids; or (iii) icodextrin were used according to the clinical needs of patients.

### Statistical analysis

SPSS version 20.0 (IBM Cor., Chicago, IL) program was used for data analysis. In the statistical analysis of the study, mean, standard deviation, frequency, and percentage values were defined. The general characteristics and demographic characteristics of the groups were determined by frequency (descriptive analysis: frequency analysis for a single variable) analysis. In pairwise comparisons; independent Samples t-Test was used to compare the mean of two independent groups. The Chi-square test was used to determine the relationship between categorical variables. A *p* ≤ 0.05 value was considered statistically significant in the entire study.

The endpoint for the peritonitis-free survival analysis of the patients was the first episode of peritonitis. Peritonitis-free survival analysis of rural and urban patient groups was calculated by Kaplan–Meier method using Log-rank test for *p* value.

During the follow-up period of patients continuing PD, in order to determine the risk factors of peritonitis at any time, a model was created with patients being the dependent variables; living environment (rural and urban) was evaluated as a univariate variable, and gender, age, marital status, living environment (rural and urban), number of people living in the same house, type of house structure, type of house heating, education level, diagnosis for diabetes mellitus, diagnosis for hypertension, PD method, history of HD, preference reason for PD, PD practitioner, presence of reserved room, and use of bathroom tape were evaluated as multivariate variables of the independent variables. Cox regression analysis was performed on the determined model. All tests were bidirectional. Confidence intervals of 5% are given for the risk of type I error. The study sample was determined as 141 using the PASS version 15 program (Kaysville, Utah) by taking hazard ratio 1.2, *α* = 0.05, power (1–*β*) = 0.90 confidence level. A total of 202 were reached. A *p* ≤ 0.05 value was considered statistically significant in the entire study.

## Results

A total of 202 PD patients were followed during 791 patient-years (mean follow-up of 3.9 years per patient). Patients experienced a total of 335 episodes of peritonitis and 0.42 episodes per patient-year of treatment. While 60 (30%) of the examined patients lived in rural areas, 142 (70%) patients lived in urban areas. Of the patients in the rural area, 29 (48.3%) were female and 31 (51.7%) were male. Of the patients in the urban area, 66 (46.5%) were female and 76 (53.5%) were male. There was no difference between the mean age of patients living in rural areas and patients living in urban areas (51.55 ± 16.117, 54.75 ± 15.528, respectively). Other characteristics are presented in Table 1.

**Table 1.** Demographic and other characteristics of people living in rural and urban areas.

		All patients (*n* = 202)		
		Rural (*n* = 60)	Urban (*n* = 142)	*p*
Gender	Female *n* (%)	29 (48.3)	66 (46.5)	0.809
	Male *n* (%)	31 (51.7)	76 (53.5)	
Age	Mean ± SD	51.55 ± 16.117	54.75 ± 15.528	0.188
Marital status	Married *n* (%)	44 (73.3)	114 (80.3)	0.351
	Single *n* (%)	16 (26.7)	28 (19.7)	
Educational status	Basic training	53 (88.3)	101 (71.1)	0.011*
	Higher education *n* (%)	7 (11.7)	41 (28.9)	
Number of people living in the same house with the patient	≥ 4 *n* (%)	49 (81.7)	100 (70.4)	0.097
	< 3 *n* (%)	11 (18.3)	42 (29.6)	
Number of people living in the same house with the patient	Mean ± SD	5.43 ± 2.849	4.67 ± 2.449	0.055
Duration of peritoneal dialysis (years)	Mean ± SD	4.47 ± 2.633	3.68 ± 2.981	0.079
House structure shape	Soil *n* (%)	41 (68.3)	2 (1.4)	0.001*
	Reinforced concrete *n* (%)	19 (31.7)	140 (98.6)	
House heating type	Stove-heated *n* (%)	57 (95)	21 (14.8)	0.001*
	Radiator heated *n* (%)	3 (5)	121 (85.2)	
Etiology of end-stage renal disease	DM *n* (%)	14 (23.3)	49 (34.5)	0.157
	HT *n* (%)	14 (23.3)	44 (31)	
	CIN *n* (%)	10 (16.3)	15 (10.6)	
	GN (%)	10 (16.3)	18 (12.7)	
	Other *n* (%)	12 (20)	16 (11.3)	
DM status	Present *n* (%)	14 (23.3)	49 (34.5)	0.136
	None *n* (%)	46 (76.7)	93 (65.5)	
HT status	Present *n* (%)	20 (33.3)	58 (40.8)	0.346
	None *n* (%)	40 (66.7)	84 (59.2)	
Method of peritoneal dialysis	IPD *n* (%)	14 (23.3)	50 (35.2)	0.102
	CAPD *n* (%)	46 (76.7)	92 (64.8)	
Is there a history of hemodialysis before peritoneal dialysis?	Yes *n* (%)	3 (5)	10 (7)	0.589
	No *n* (%)	57 (95)	132 (93)	
Reason for peritoneal dialysis Preference	Preference *n* (%)	47 (78.3)	127 (89.4)	0.037*
	Necessity *n* (%)	13 (21.7)	15 (10.6)	
Peritoneal dialysis practitioner	In person *n* (%)	41 (68.3)	100 (70.4)	0.867
	Assistant *n* (%)	19 (31.7)	42 (29.6)	
Is there a room dedicated for peritoneal dialysis?	Yes *n* (%)	33 (55)	100 (70.4)	0.035*
	No *n* (%)	27 (45)	42 (29.6)	
Is bath tape used?	Yes *n* (%)	52 (86.7)	123 (86.6)	0.993
	No *n* (%)	8 (13.3)	19 (13.4)	
Peritonitis experience (at least once *vs*. never)	Experienced (at least 1 peritonitis) *n* (%)	45 (75)	80 (56.3)	0.013*
	Did not experienced (without peritonitis) *n* (%)	15 (25)	62 (43.7)	
Peritonitis rates (episodes per patient/year)	Mean ± SD	0.61 ± 0.749	0.49 ± 0.737	0.256
Time to the first peritonitis episode (month)	Mean ± SD	24.33 ± 20.905	23.33 ± 25.173	0.301

* *p* < 0.05 value was considered statistically significant.

IPD: instrumented peritoneal dialysis; CAPD: continuous ambulatory peritoneal dialysis; DM: diabetes mellitus; HT: hypertension; GN: glomerulonephritis; CIN: Chronic interstitial nephritis.

Bacterial peritonitis was detected in 287 (85.7%) and fungal peritonitis was detected in 10 (3%) of the patients, while no growth or contamination was detected in the cultures of the remaining 38 cases (11.3%). When the causative microorganisms of bacterial peritonitis were examined, Methicillin-sensitive coagulase-negative staphylococci (MSCNS) were found in the first place with 30.9%, and methicillin-resistant coagulase-negative staphylococci (MRCNS) were found in the second place with 17.1%. These were followed by *Staphylococcus aureus*, *Escherichia coli*, and *enterococci*, respectively. The most common cause of fungal peritonitis was found to be *Candida parapsilosis* and *Candida albicans*. Mycobacterium tuberculosis was detected in only two patients. There was no difference in cultural results between those living in rural and urban areas.

The proportion of those who never experienced peritonitis was higher in the urban area (43.7 *vs.* 25%) (Table 1). In addition to this, there were differences between the groups, such as education level, the structure of the house they lived in, the way the house was heated, the reason for the preference for PD, and the presence of a room reserved for PD. There was no statistically significant difference between the time to the first peritonitis attack (rural = 24.33 ± 20.905 months and urban = 23.33 ± 25.173 months) (*p* = 0.301) and patient peritonitis rates (rural = 0.61 ± 0.749 and urban = 0.49 ± 0.737) (*p* = 0.256) (Table 1).

When we examine only the patients who experienced peritonitis living in urban and rural areas; the peritonitis rate of the urban group was 0.86 episodes per year, while the peritonitis rate of the patients living in rural areas was 0.82 episodes per year (*p* = 0.772). The differences between rural and urban residents with peritonitis were educational status, type of heating structure of the house, having a separate room for PD, and the rate of PD out of necessity (Table 2).

**Table 2.** Demographic and other characteristics of patients with peritonitis who are living in urban and rural areas.

		Rural (*n* = 45)	Urban (*n* = 80)	*p*
Gender	Female *n* (%)	24 (53.3)	43 (53.8)	0.964
	Male *n* (%)	21 (46.7)	37 (46.2)	
Age	Mean ± SD	50.36 ± 17.361	54.33 ± 15.173	0.185
Marital status	Married *n* (%)	31 (68.9)	63 (78.8)	0.281
	Single *n* (%)	14 (31.1)	17 (21.2)	
Educational status	Basic education	41 (91.1)	58 (72.5)	0.020*
	Higher education *n* (%)	4 (8.9)	22 (27.5)	
Number of people living in the same house with the patient	≥4 *n* (%)	36 (80)	56 (70)	0.292
	<3 *n* (%)	9 (20)	24 (30)	
Number of people living in the same house with the patient	Mean ± SD	5.56 ± 3.086	4.83 ± 2.671	0.168
Duration of peritoneal dialysis (years)	Mean ± SD	5 ± 2.697	4.25 ± 3.347	0.201
House structure shape	Soil *n* (%)	31 (68.9)	0	0.001*
	Reinforced concrete *n* (%)	14 (31.1)	80 (100)	
House heating type	Stove-heated *n* (%)	43 (95.6)	9 (11.2)	0.001*
	Radiator heated *n* (%)	2 (4.4)	71 (88.8)	
Etiology of end-stage renal disease	DM *n* (%)	7 (15.6)	24 (30.0)	0.264
	HT *n* (%)	12 (26.7)	25 (31.2)	
	CIN *n* (%)	7 (15.6)	8 (10.0)	
	GN (%)	10 (22.2)	14 (17.5)	
	Other *n* (%)	9 (20)	9 (11.2)	
DM status	Present *n* (%)	7 (15.6)	24 (30.0)	0.087
	None *n* (%)	38 (84.4)	56 (70.0)	
HT status	Present *n* (%)	15 (33.3)	32 (40.0)	0.565
	None *n* (%)	30 (66.7)	48 (60.0)	
Method of peritoneal dialysis	IPD *n* (%)	8 (17.8)	29 (36.2)	0.041*
	CAPD *n* (%)	37 (82.2)	51 (63.7)	
Is there a history of hemodialysis before peritoneal dialysis?	Yes *n* (%)	2 (4.4)	5 (6.2)	0.673
	No *n* (%)	43 (95.6)	75 (93.5)	
Reason for peritoneal dialysis Preference	Preference *n* (%)	36 (80)	72 (90)	0.117
	Necessity *n* (%)	9 (20)	8 (10)	
Peritoneal dialysis practitioner	In person *n* (%)	31 (68.9)	59 (73.8)	1.000
	Assistant *n* (%)	14 (31.1)	21 (26.2)	
Is there a room dedicated for peritoneal dialysis?	Yes *n* (%)	26 (57.8)	61 (76.2)	0.031*
	No *n* (%)	19 (42.2)	19 (23.8)	
Is bath tape used?	Yes *n* (%)	38 (84.4)	68 (85)	0.934
	No *n* (%)	7 (15.6)	12 (15)	
Peritonitis rates (episodes per patient/year)	Mean ± SD	0.8240 ± 0.762	0.8665 ± 0.799	0.772

* *p* < 0.05 value was considered statistically significant.

IPD: instrumented peritoneal dialysis; CAPD: continuous ambulatory peritoneal dialysis; DM: diabetes mellitus; HT: hypertension; GN: glomerulonephritis; CIN: chronic interstitial nephritis.

**Figure 1.**
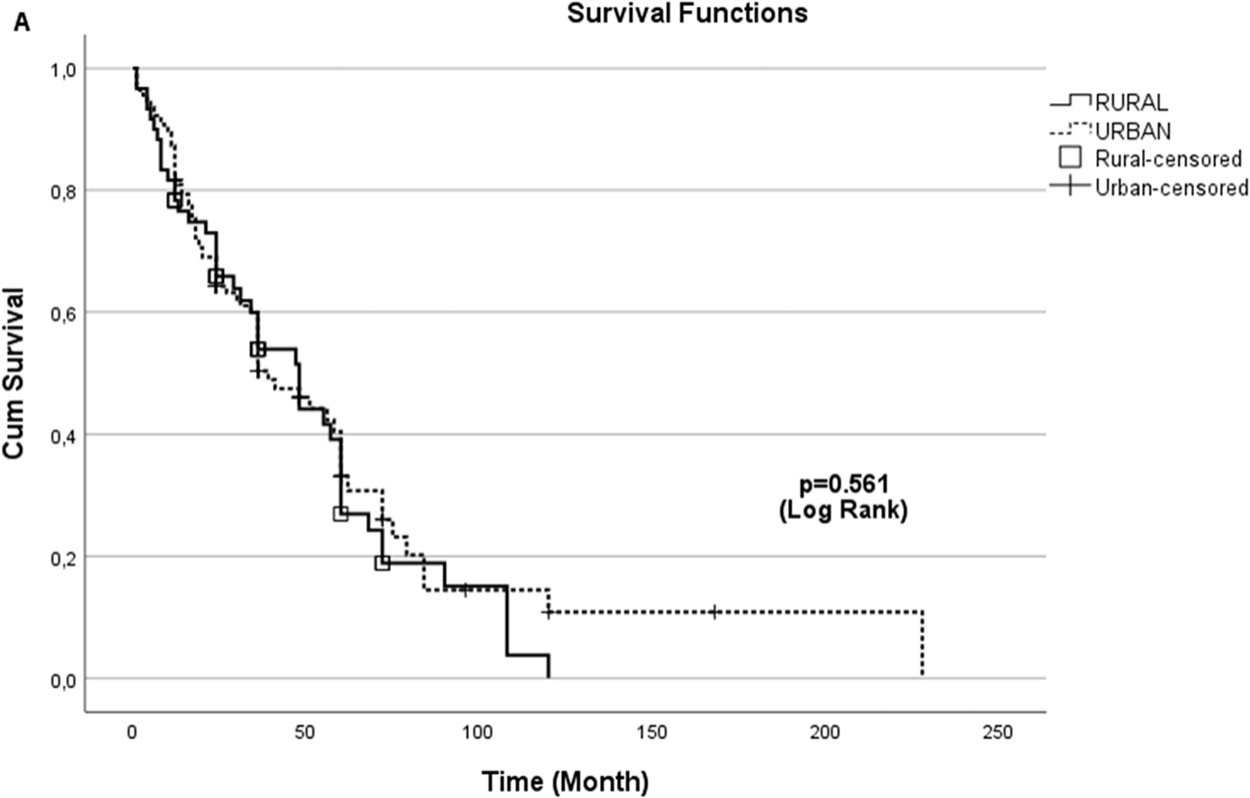
Kaplan–Meier analysis of peritonitis-free survival: comparison of rural and urban patient groups.

In the analysis made by Kaplan–Maier method between rural and urban groups; no statistically significant difference was found for peritonitis-free survival (Log-rank *p* = 0.561) (Figure 1(A)).

**Figure 2.**
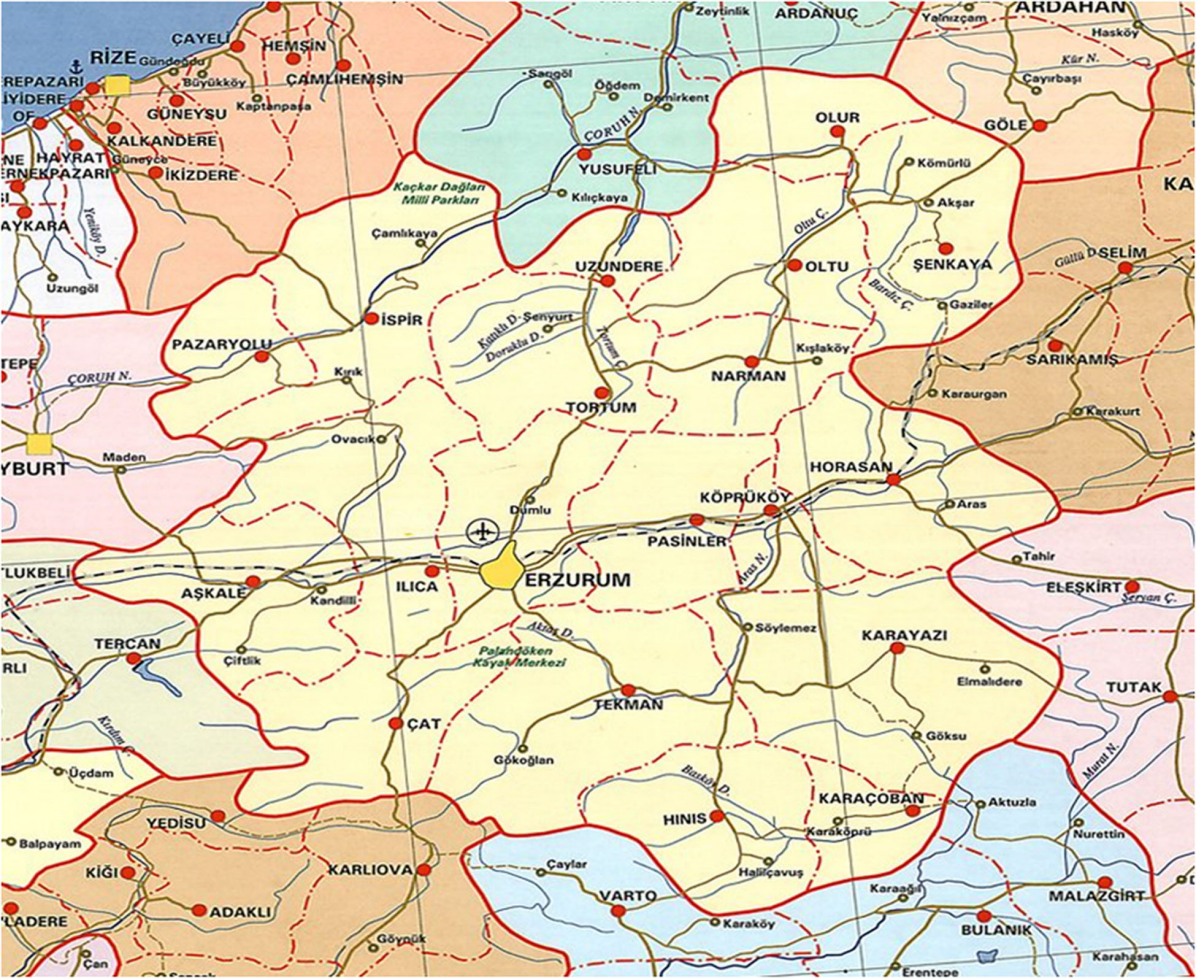
Erzurum center, districts, and neighboring provinces map (the only peritoneal center in this region (
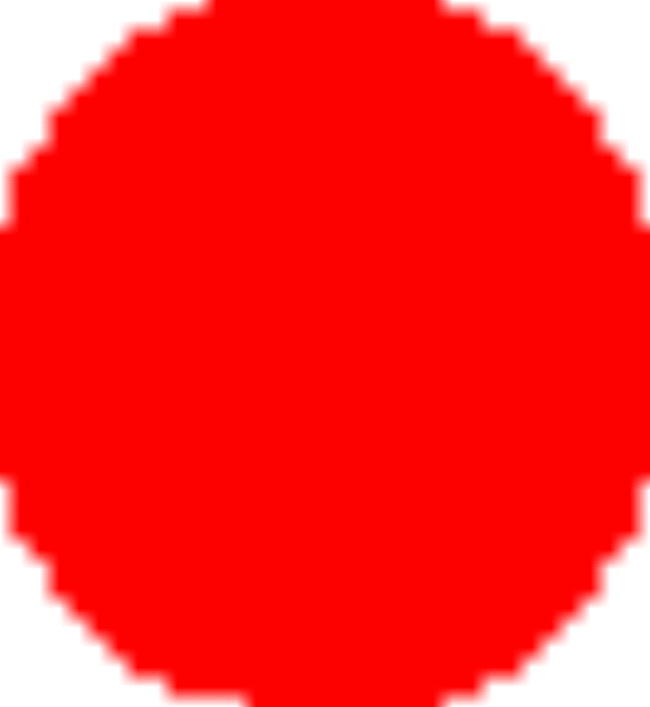
, 
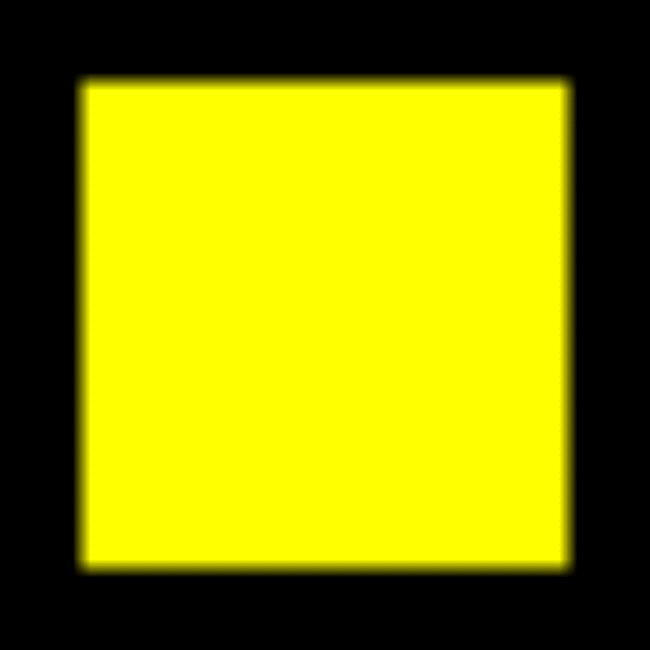
; urban areas).

By univariate Cox regression analysis, living in rural areas does not increase the risk of peritonitis (Table 3A). When we evaluated the factors affecting peritonitis in PD in Multivariate Cox-regression analysis, it was observed that social and demographic characteristics such as living in a rural environment, having a separate room for PD, the type of structure of the house, the heating type of the house, educational status, number of people living in the house, marital status did not have an effect (Table 3B).

**Table 3.** Results of Cox-regression analysis of peritoneal dialysis patients affecting peritonitis.

		*β*	SE*_β_*	Wald	sd	*p*	eβ = OR	eβ = 95% confidence interval for OR	
								Bottom	Upper
A									
Living environment	Rural	−0.002	0.189	0.000	1	0.993	0.998	0.689	1.446
	Urban	Reference							
B									
Gender	Female	Reference							
	Male	0.030	0.207	0.022	1	0.883	1.031	0.687	1.546
Age		−0.006	0.007	0.792	1	0.374	0.994	0.980	1.008
Marital status	Married	Reference							
	Single	−0.006	0.253	0.001	1	0.981	0.994	0.606	1.631
Living environment	Rural	0.199	0.354	0.317	1	0.574	0.819	0.409	1.641
	Urban	Reference							
Number of people living in the same house	≥ 4	Reference							
	< 3	−0.236	0.237	0.988	1	0.320	0.790	0.496	1.258
House structure shape	Soil	0.525	0.337	2.422	1	0.120	1.691	0.873	3.276
	Reinforced concrete	Reference							
House heating type	Stove-heated	Reference							
	Radiator heated	0.092	0.337	0.075	1	0.785	0.912	0.471	1.766
Educational status	Basic training	0.164	0.262	0.390	1	0.533	0.849	0.508	1.420
	Higher Education	Reference							
DM status	None	Reference							
	Present	0.219	0.227	0.928	1	0.335	1.245	0.797	1.944
HT status	None	Reference							
	Present	0.297	0.229	1.686	1	0.194	1.346	0.860	2.107
Method of peritoneal dialysis	IPD	Reference							
	CAPD	0.102	0.217	0.219	1	0.639	1.107	0.723	1.695
Hemodialysis history	No	Reference							
	Yes	0.332	0.509	0.425	1	0.515	1.394	0.514	3.782
Reason for peritoneal dialysis preference	Preference	Reference							
	Necessity	0.083	0.348	0.057	1	0.811	1.087	0.550	2.147
Peritoneal dialysis practitioner	In-person	Reference							
	Assistant	0.412	0.224	3.378	1	0.066	1.509	0.973	2.341
Dedicated room	No	Reference							
	Yes	−0.136	0.216	0.394	1	0.530	0.873	0.572	1.334
Bath tape	No	Reference							
	Yes	0.318	0.292	1.189	1	0.276	1.374	0.776	2.434

*p* < 0.05 value was considered statistically significant.

DM: Diabetes mellitus; HT: hypertension; IPD: instrumented peritoneal dialysis; CAPD: continuous ambulatory peritoneal dialysis.

A: Results of Cox-regression analysis for the univariate model of peritoneal dialysis patients: effect of living in a rural or urban area on peritonitis.

B: Results of Cox-regression analysis for the multivariate model of peritoneal dialysis patients: factors affecting peritonitis.

## Discussion

With the Progressive Kidney Health Initiative’s report in 2019, there is a new push for home dialysis in the United States, with the goal that 80% of new patients with kidney failure will receive home dialysis or kidney transplantation by 2025 [7]. A study by American nephrologists found that distances of >50 km between the patient’s residence and the dialysis facility had a significant impact on the choice of modality. With the ability to monitor treatments remotely and make monthly clinic visits, a home dialysis is a convenient option for patients who do not live near a dialysis unit. This is especially valid in rural areas. Studies have reported that peritoneal patients mostly live in rural areas [8–10]. Therefore, it was important to evaluate the relationship between PD and peritonitis, the most important cause of mortality and morbidity in rural areas. This study is one of the rare studies performed on how the life differences of PD patients in rural areas will affect peritonitis. Our peritonitis patient-year rate at our center was 0.42. There is great variation in the rates of PD peritonitis between different centers and countries. Reported rates range from 0.06 to 1.66 episodes/patient-year [4]. Our PD peritonitis culture-negative rate was per the ISPD recommended rate of less than 15% of acceptable culture-negative peritonitis episodes [2].

PD patients living in rural areas had a similar rate of peritonitis and a lower rate of patients without peritonitis. Studies have generally emphasized the relationship between living far from the PD center and peritonitis [5,11]. It is reported that living far from the PD center increases the risk of peritonitis. In addition, some studies estimated that the high rate of peritonitis in those living close to the PD center may have reflected worse hygiene conditions in urban environments [12]. Living far from the PD center has been associated with the risk of peritonitis in a short span of time [5]. The low rate of patients without peritonitis in rural areas in our study supports this. Manish M Sood et al. in the study they performed, reported that there was no significant difference in terms of peritonitis among aboriginals (61 patients in total) living in rural and urban areas [13]. The low number of cases may not have revealed the difference in this regard. In studies conducted on this subject, it may be possible to obtain contradictory results due to geographical differences, ethnic origin, and socio-cultural and economic differences in urban and rural areas. There was no ethnic difference in our study. All our patients who experienced peritonitis were given standard training by the same PD training nurse, and our training was repeated after each peritonitis episode. In addition, the education level of PD patients living in rural areas was lower than patients living in urban areas. For this reason, standard training may not be sufficient for patients living in rural areas. Compared to PD patients from urban settings, PD patients living in rural areas had a higher rate of developing at least 1 peritonitis episode and a lower rate of never developing peritonitis. This may be due to repetitive training. It is known that PD education in patients is especially important for peritonitis [14]. In addition, developing and increasing educational tools and trainers may contribute to the decrease in peritonitis rates compared to previous years [15]. It was found that only 74% of PD patients performed the PD procedure correctly in terms of infection prophylaxis. Based on this result and an assessment of the knowledge of 353 patients who completed a questionnaire, the authors estimated that 29% of patients needed retraining of the PD replacement technique. In addition, an observational study in Italy showed that hospitals that provided retraining to PD patients had lower rates of peritonitis than those that did not [16,17]. Therefore, we believe that it would be beneficial to individualize PD training according to the patient. In the study performed by Martin LC et al. [12], they reported that the training level of the patients may be an important risk factor for peritonitis. In addition, there is no evidence that the risk of peritonitis is related to when, where, how and for how long PD patients should be educated. PD patient education supports that PD can be delivered in any way that is compatible with locally available resources and individualized to the needs of the patient [18].

In the microbiological examination of our patient group, the most common agent was Coagulase-negative Staphylococci. It was similar to the studies performed [2,19]. In addition, publications are reporting that *Staphylococcus aureus* is the most common factor in patients living far from the PD center [5,11]. Nasal colonization has been reported to be an important factor, and the use of mupirocin has been shown to reduce the rate of peritonitis [5,20,21]. The reason why it was not in the top ranks in our study may be that nasal colonization was investigated and treated at the beginning of PD.

## Conclusion

In PD, social life and demographic differences of rural residents are not a parameter affecting the rate of peritonitis in our region. It is not the right approach for both the physician and the patient to be reluctant in the choice of PD due to the concern of peritonitis in rural areas.

## Study limitations

There were strengths and limitations in our study. Being a large PD center in this region with over 30 years of PD experience in the east of Turkey, presenting the experiences of a center with a different geographical location to the literature, homogeneity of our patient group, and making social contributions were our strengths. The limitations of our study were that our patient group was limited.

## Data Availability

The datasets generated during and/or analyzed during this study are not publicly available but are available from the corresponding author on reasonable request.
